# 624. Building Regional Capacity for Firework Injury Prevention: Early Outcomes of a Framework-based Collaborative

**DOI:** 10.1093/jbcr/irag033.450

**Published:** 2026-04-06

**Authors:** Che R Ochtli, Kim Zaky, Syed F Saquib

**Affiliations:** UCI Health Regional Burn Center, Orange, CA; UCI Health Regional Burn Center, Orange, CA; University of California Irvine, Orange, CA

## Abstract

**Introduction:**

Firework-related burns during July celebrations remain a predictable seasonal source of injury in the United States. In 2022, the American Burn Association (ABA) released its Burn Prevention Framework to guide prevention programming, though few published applications of this framework exist.

**Methods:**

In 2024 a multi-organization collaborative was established comprising representation from healthcare institutions, fire service, burn foundations, and school districts. Stakeholder activities were mapped to the ABA Burn prevention framework:

1) Assess the problem- Institutional and county after-action reports were reviewed. Data guided the identification of fireworks as a recurring seasonal mechanism of injury, prevalence, severity, and geographic clustering.

2) Target the population- high-risk zip codes, schools, and firework distribution points were prioritized.

3) Identify resources – partnerships leveraged existing networks across communities and disciplines to increase burn prevention capacity.

4) Develop a plan – a unified prevention message was created each year with consistent branding and culturally tailored materials;

5) Implement and evaluate – outreach included media, print, and evergreen digital deliverables, institutional and county reporting, and collective accountability.

**Results:**

In Year 1, the collaborative engaged 15 organizations, held 4 meetings, and created 1 evergreen resource (“Firework Safety Basics”) cobranded with 12 fire departments. Data were reported from 10/25 hospitals and 6/94 urgent cares. By Year 2, engagement grew to 22 organizations and 9 meetings, with translations of the resource into 3 languages. Data were reported from 13/25 hospitals and 12/94 urgent cares, with injuries dropping from 66 to 44. New initiatives included integration into “Stop the Bleed” courses (697 students) and sports arena messaging reaching thousands. Tracking and partner feedback confirmed adoption of the unified message across organizations.

**Conclusions:**

Application of the ABA Burn Prevention Framework to firework prevention was feasible and effective. Early outcomes demonstrate its utility for structuring outreach and unified messaging around seasonal, high-risk mechanisms of injury. It also provides proof of concept for continual refinement and expansion of prevention activities.

**Applicability of Research to Practice:**

This initiative demonstrates that the ABA Framework can be operationalized in real-world, mechanism-specific prevention efforts, offering a replicable model for burn centers and community partners to increase regional capacity.

**Funding for the study:**

N/A.

